# Supplementary material for: New, Eco-Friendly Method for Synthesis of 3-Chlorophenyl and 1,1′-Biphenyl Piperazinylhexyl Trazodone Analogues with Dual 5-HT1A/5-HT7 Affinity and Its Antidepressant-like Activity
Source: Molecules. 2022 Oct 26;27(21):7270. doi: 10.3390/molecules27217270 (PMC9658223; doi:10.3390/molecules27217270)
Supplement: Supplementary file 1 [file molecules-27-07270-s001.zip › molecules-1968218-supplementary.pdf]

Supplementary information

Characterization of 2-(6-(4-(3-chlorophenyl)piperazin-1-yl)hexyl)-[1,2,4]triazolo[4,3-*a*]pyridin-3(*2H*)-one hydrochloride (7a·HCl) 2

Characterization of 2-(6-(4-(2-phenylphenyl)piperazin-1-yl)hexyl)-[1,2,4]triazolo[4,3-*a*]pyridin-3(*2H*)-one hydrochloride (7b·HCl) 4

ADMET predictor parameters for the designed compounds and reference (trazodone) 6

Data for functional research 8

Characterization of 2-(6-(4-(3-chlorophenyl)piperazin-1-yl)hexyl)-[1,2,4]triazolo[4,3-*a*]pyridin-3(*2H*)-one hydrochloride (7a·HCl)

UPLC-MS:

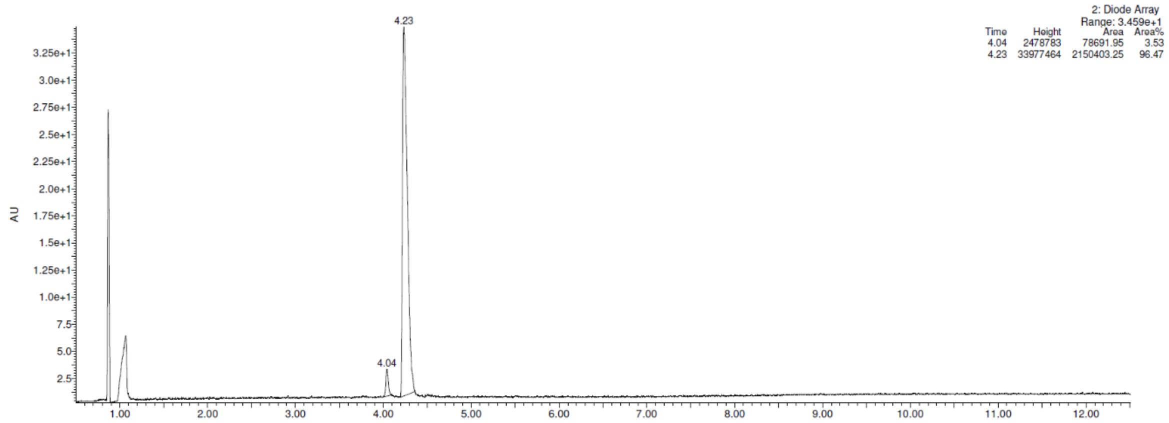

Figure S1. UPLC chromatogram of **7a·HCl**.

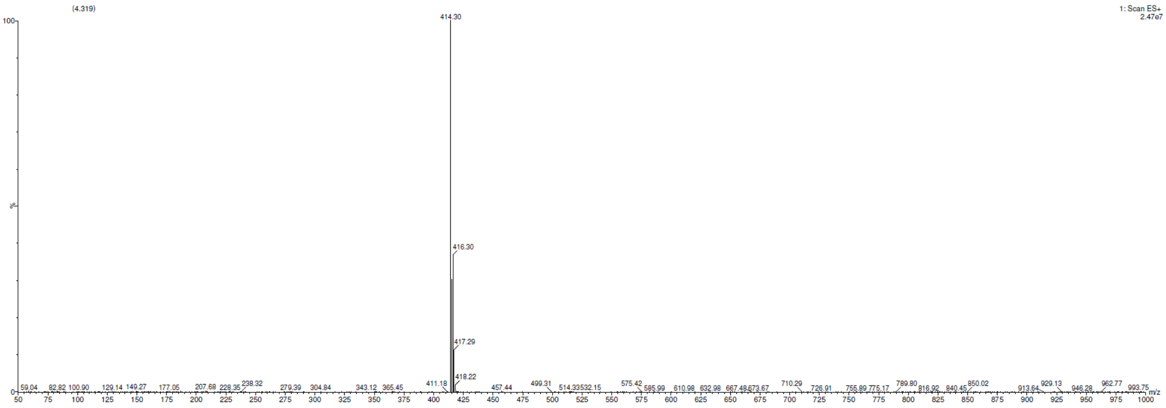

Figure S2. MS spectrum of **7a·HC**l.

^{1}H NMR:

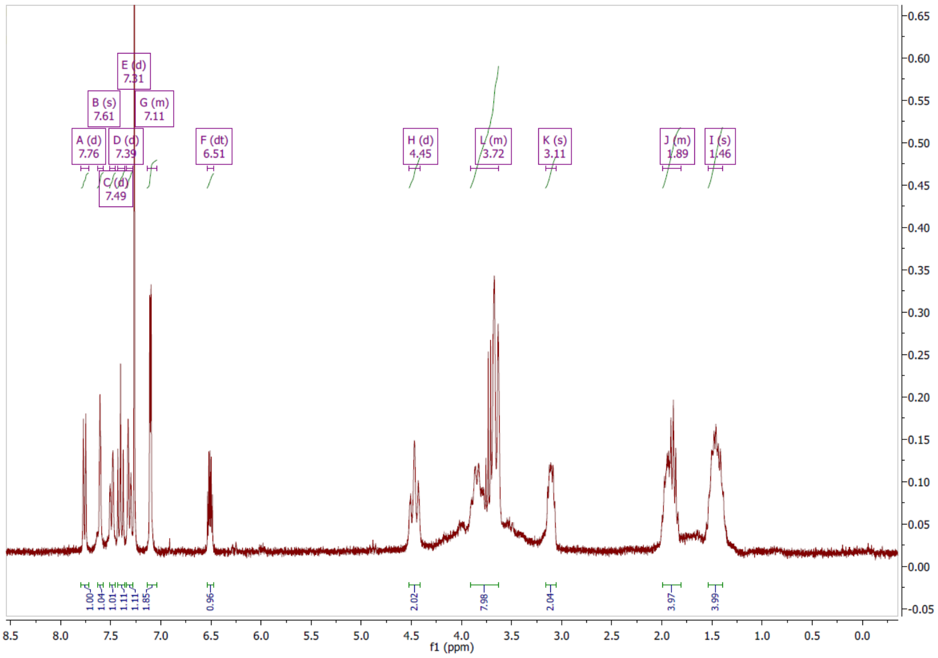

Figure S3. ^{1}H NMR of **7a·HCl**.

^{13}C NMR:

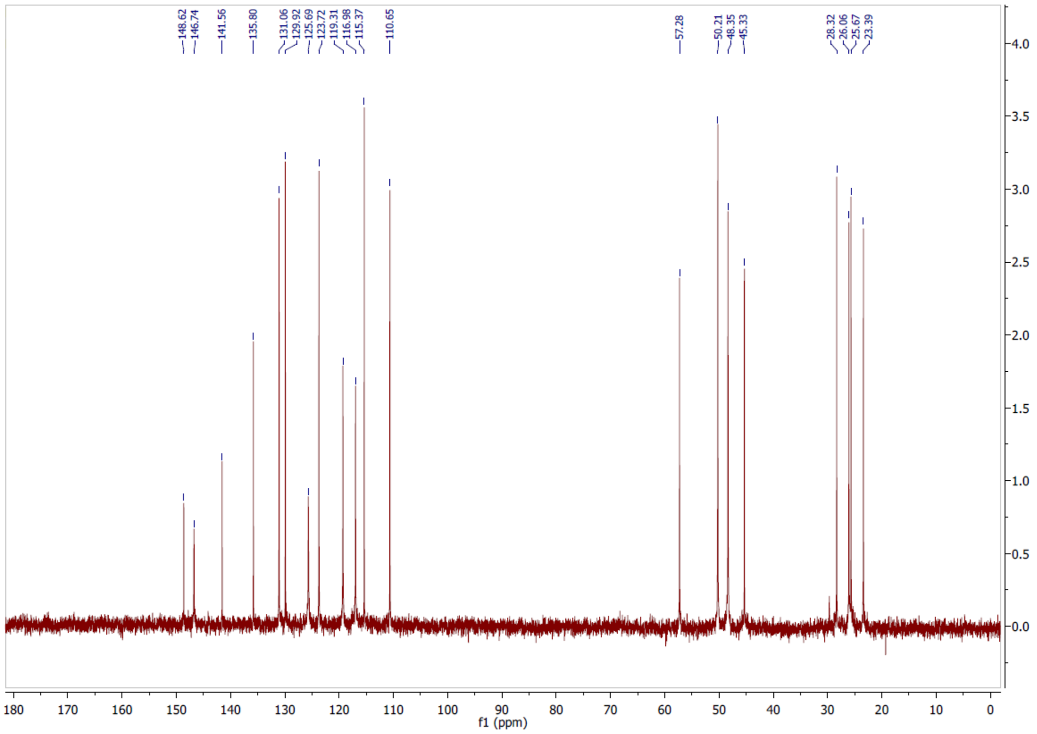

Figure S4. ^{13}C NMR of **7a·HCl**.

Characterization of 2-(6-(4-(2-phenylphenyl)piperazin-1-yl)hexyl)-[1,2,4]triazolo[4,3-*a*]pyridin-3(*2H*)-one hydrochloride (7b·HCl)

UPLC-MS:

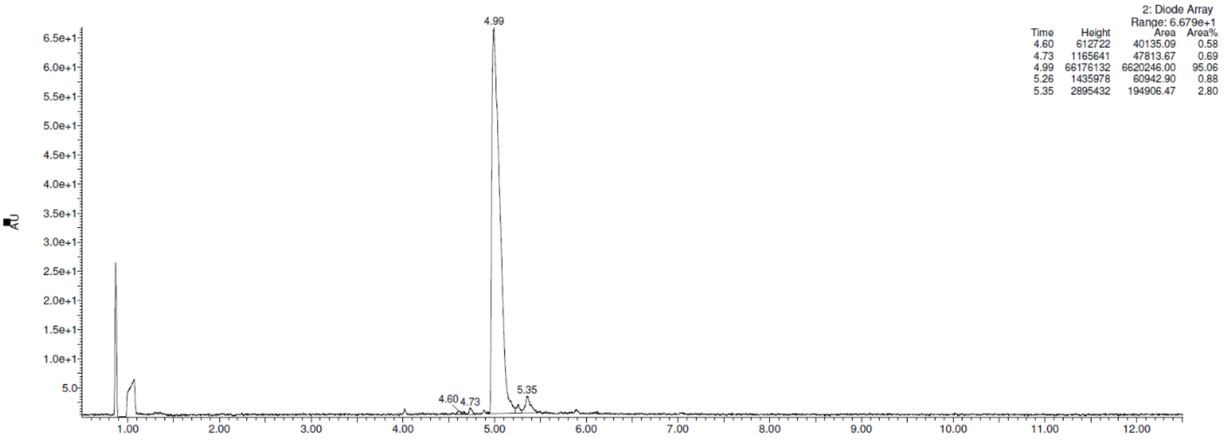

Figure S5. UPLC chromatogram of **7b·HCl**.

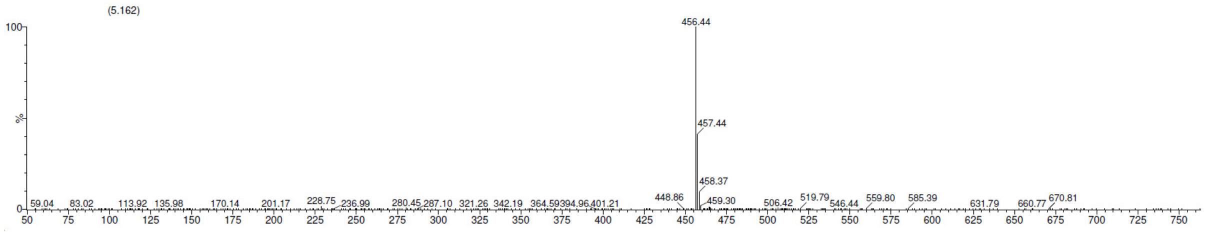

Figure S6. MS spectrum of **7b·HCl**.

^{1}H NMR:

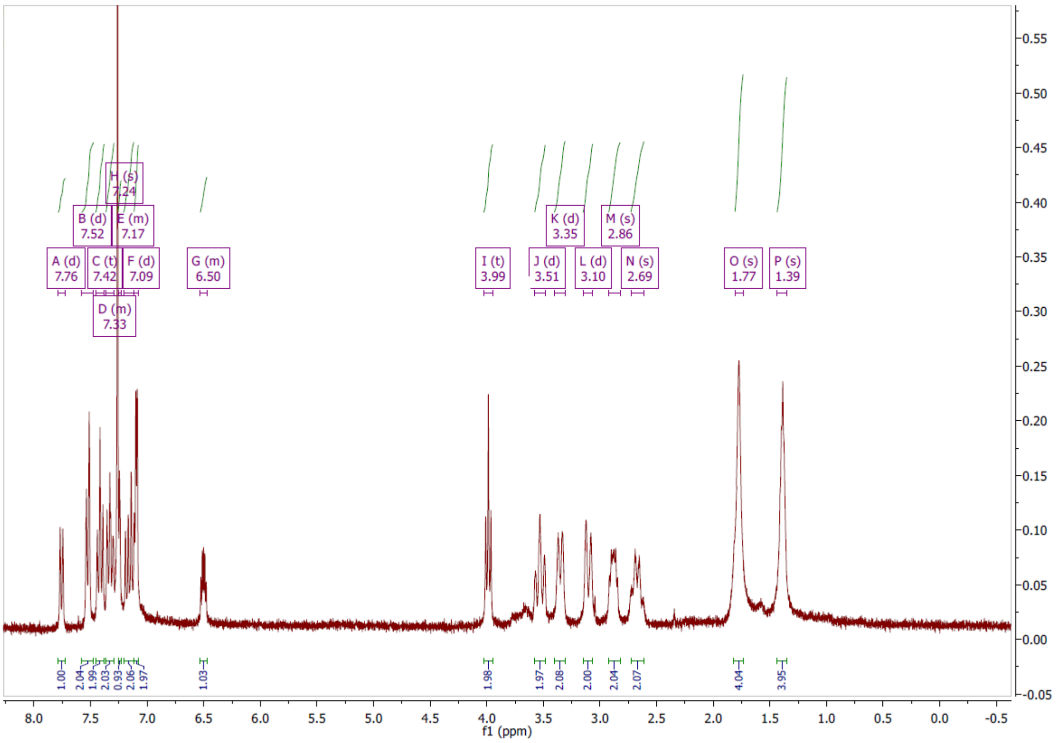

Figure S7. ^{1}H NMR of **7b·HCl**.

^{13}C NMR:

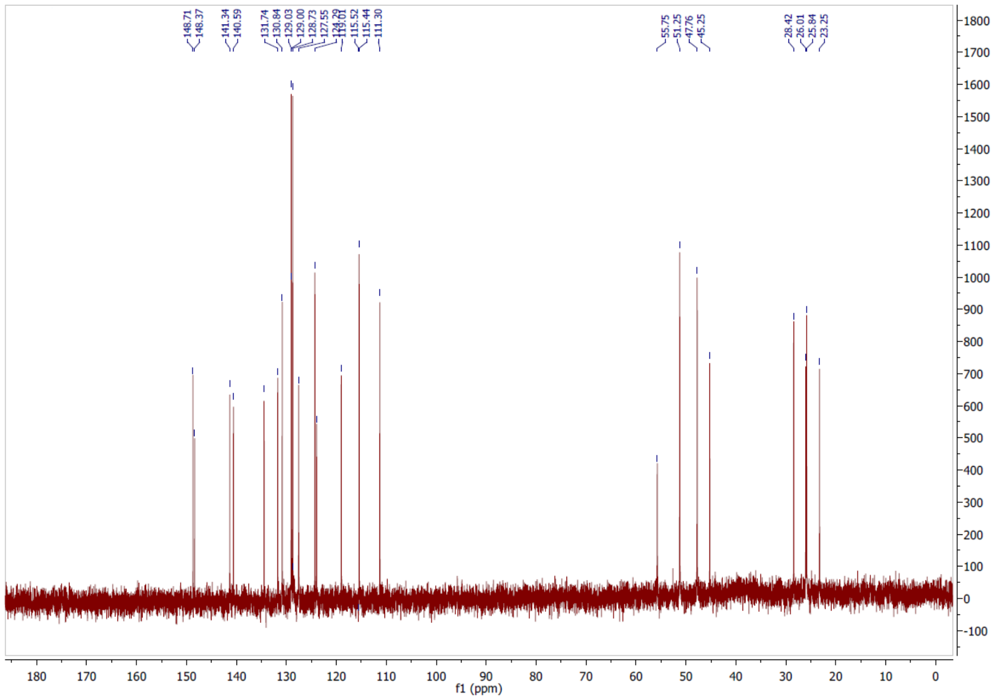

Figure S8. ^{13}C NMR of **7b·HCl**.

ADMET predictor parameters for the designed compounds and reference (trazodone)

Table S1. ADMET predictor parameters for the designed compounds and reference (trazodone).

	ADME / compound	trazodone	7a	7b
Absorption	Lipinski's Rule of 5[a]	0	0	1
	Absorption Risk[b]	1.0	1.0	2.8
	Water solubility (mg/mL)	0.024	0.022	0.003
	Solubility in simulated gastric fluid (mg/mL)	6.171	5.415	3.846
	Effective human jejunal permeability (cm/s x 10^4)	5.002	3.793	1.983
	P-glycoprotein substrate	No (80%)	No (48%)	Yes (66%)
	P-glycoprotein inhibitor	No (53%)	Yes (66%)	Yes (88%)
	BCRP substrate	No (95%)	No (95%)	No (67%)
	BCRP inhibitor	No (87%)	No (76%)	Yes (56%)
Distribution	Volume of distribution (L/kg)	2.652	4.245	2.547
	% unbound to blood plasma proteins	6.605	5.579	3.256
	Penetration of Blood Brain Barrier	High (99%)	High (99%)	High (99%)
	Log of the Brain/Blood partition coef.	0.433	0.711	0.377
Metabolism	CYP_Risk[c]	0.534	1.227	2.763
	CYP 2C19 inhibitor	Yes (23%)	Yes (26%)	Yes (19%)
	CYP 2C19 substrate	No (80%)	No (85%)	No (88%)
	CYP 2D6 inhibitor	No (95%)	No (65%)	Yes (44%)
	CYP 2D6 substrate	Yes (55%)	Yes (59%)	Yes (43%)
	CYP 3A4 inhibitor	No (76%)	No (67%)	No (65%)
	CYP3A4 substrate	Yes (98%)	Yes (98%)	Yes (98%)
Excretion	Clearance mechanism is metabolism	Yes (99%)	Yes (99%)	Yes (99%)
	OCT2 substrate	No (91%)	No (91%)	No (91%)
Toxicity	Mutagenic chromosomal aberrations	No (61%)	No (75%)	No (96%)
	Risk of mutagenicity[d]	1.8	0.6	0

	Risk of toxicity[e]	2.184	2.000	2.500
	Reproductive / developmental toxicity	No (91%)	No (91%)	No (72%)
	Blocks the hERG potassium channel	Yes (82%)	Yes (99%)	Yes (95%)
	Affinity to the hERG K^+ channel	5.592	6.055	6.180
	Skin sensitization	Yes (99%)	Yes (99%)	Yes (93%)

[a]A score indicating the number of potential problems a compound is expected to have with passive oral absorption.[b]A score in the 0-8 range indicating the number of potential oral absorption problems a compound is likely to have. [c]Risk connected with P450 oxidation: a score in the 0-6 range indicating the number of potential problems a compound might have due to metabolism by one or more of five major cytochrome P450s. [d]A score in the 0-5.4 range that is a weighted sum of the number of 'Positive' predictions by the TOX_MUT_* models (Ames mutagenicity in 5 strains of Salmonella typhimurium + an NIHS Ames panel) with or without microsomal activation. [e]A score in the 0-6 range indicating the number of potential toxicity problems a compound might have.

Data for functional research

2-(6-(4-(3-chlorophenyl)piperazin-1-yl)hexyl)-[1,2,4]triazolo[4,3-*a*]pyridin-3(2*H*)-one hydrochloride (7a·HCl)

Table S2. Change in cAMP concentration in dependence on **7a·HCl** concentration, for 5-HT_7R.

log [M]	7a·HCl [%]		
-5	98.9	90	93
-5.6	102.5	96.4	101.1
-6.2	54.4	67.3	63
-6.8	20.6	21	8.5
-7.4	10.9	9.4	-7.2
-8	13.7	-4.5	5.9
-8.6	19.5	1	0.7
-9.2	4.4	-0.1	-4.3

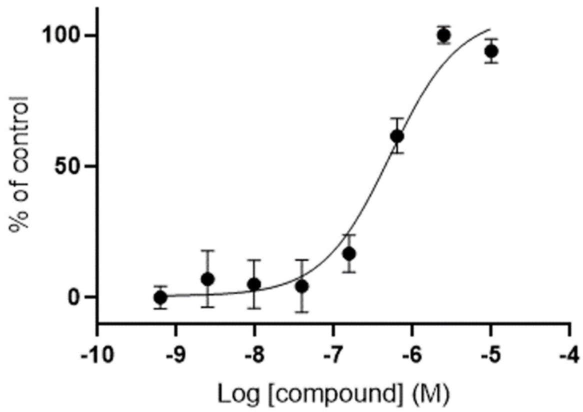

Figure S9. cAMP concentration change response curve as a function of **7a·HCl** concentration, for 5-HT_7R.

2-(6-(4-(2-phenylphenyl)piperazin-1-yl)hexyl)-[1,2,4]triazolo[4,3-*a*]pyridin-3(2*H*)-one hydrochloride (7b·HCl)

Table S3. Change in cAMP concentration in dependence on **7b·HCl** concentration, for 5-HT_{1A}R.

log [M]	7b·HCl [%]		
-5	35.6	47.8	49.5
-5.6	75	65.2	87.5
-6.2	94.1	92.7	95.5
-6.8	99.1	83.4	98.2
-7.4	107.3	98.6	91
-8	113	109.6	95
-8.6	111	82.3	103.5
-9.2	112.2	100.6	83.3

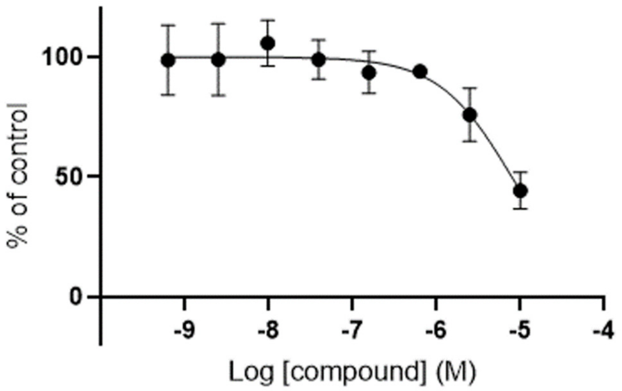

Figure S10. cAMP concentration change response curve as a function of **7b·HCl** concentration, for 5-HT_{1A}R.

Table S4. Change in cAMP concentration in dependence on **7b·HCl** concentration, for 5-HT_7R.

log [M]	7b [%]		
-5	103.8	106.3	89.9
-5.6	101.8	95.6	101.8
-6.2	98.7	98.8	95.2
-6.8	68.6	62.8	69.4
-7.4	8.2	16.2	25.3
-8	6.8	-2.9	-3.9
-8.6	7.9	-1.1	-3.4
-9.2	2.8	12.4	-0.3

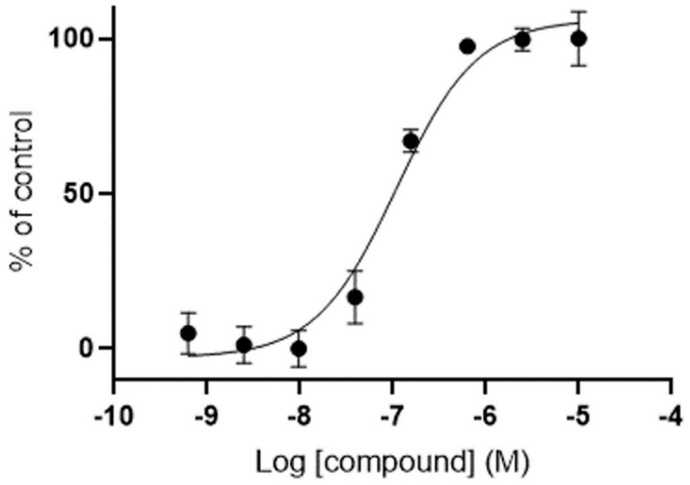

Figure S11. cAMP concentration change response curve as a function of **7b·HCl** concentration, for 5-HT_7R.
